# Exploring Divergent Electrodiagnostic and Sonographic Findings in Patients With Suspected Carpal Tunnel Syndrome: Role of Median Nerve Cross‐Sectional Area

**DOI:** 10.1002/jcu.23981

**Published:** 2025-04-09

**Authors:** Nathan J. Savage, John S. McKell

**Affiliations:** ^1^ Department of Physical Therapy Winston‐Salem State University Winston‐Salem North Carolina USA; ^2^ Department of Physical Therapy McKell Therapy Group, LLC Orem Utah USA

**Keywords:** carpal tunnel syndrome, diagnostic accuracy, electrodiagnostic testing, median neuropathy, sonography, ultrasound imaging

## Abstract

**Objectives:**

Evaluate electrophysiologic and sonographic findings in patients with suspected carpal tunnel syndrome (CTS) that had divergent electrodiagnostic (EDX) and ultrasound imaging (USI) diagnoses of CTS.

**Methods:**

Retrospective analysis of 665 limbs from patients who underwent EDX testing and USI. MANOVA, Chi Square, and correlations were used to analyze electrophysiologic and sonographic variables in limbs with divergent findings. CTS diagnosis was determined using EDX‐ and USI‐based classification systems, stratified by median nerve cross‐sectional area (CSA) cutoff values producing large conclusive or small questionable shifts in diagnostic probability.

**Results:**

The proportion of limbs with a USI diagnosis of CTS but normal electrophysiologic findings was 17% and 35% when considering CSA cutoff values producing large conclusive and small questionable shifts in diagnostic probability, respectively. These limbs had significantly slower median sensory and motor latencies and larger distal and delta CSA compared to limbs with concordant findings. Conversely, the proportion of limbs with an EDX diagnosis of CTS but normal sonographic findings was 6% and 35% when considering CSA cutoff values producing large conclusive and small questionable shifts in diagnostic probability, respectively. These limbs had faster median motor latency, larger proximal and smaller distal and delta CSA compared to limbs with concordant findings.

**Conclusions:**

Median nerve CSA proved to be the most reliable and clinically meaningful factor in limbs with divergent EDX and USI diagnoses of CTS regardless of diagnostic accuracy threshold. These results underscore the importance of integrating EDX testing and USI in patients with suspected CTS, particularly in cases with inconclusive or conflicting findings.

## Graphical abstract

Evaluate electrophysiologic and sonographic findings in hands with divergent electrodiagnostic and ultrasound imaging examinations in patients with suspected carpal tunnel syndrome. The median nerve cross‐section area was the most reliable and clinically meaningful factor in hands with divergent electrodiagnostic and ultrasound imaging examination findings regardless of diagnostic accuracy threshold.

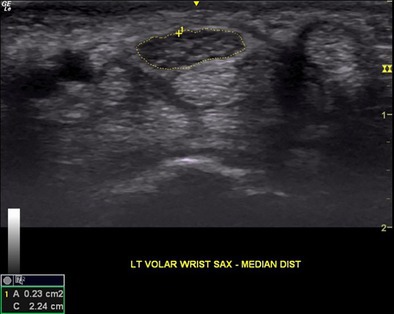

## 1 Introduction

Median neuropathy at or near the wrist, commonly described as carpal tunnel syndrome (CTS), is the most common entrapment neuropathy with an incidence of around 5% in the general population of the United States and associated costs approaching $5 billion/year [1, 2]. Although CTS is a clinical diagnosis with patients complaining of pain, paresthesia, stiffness, and swelling in the wrist and hand region, additional diagnostic tests are routinely utilized to inform the diagnosis in patients with suspected CTS [1]. Electrodiagnostic (EDX) testing, comprised of peripheral nerve conduction testing and needle electromyography, is considered by many clinicians and researchers the gold standard test for detecting neuropathic changes in the peripheral nervous system [3, 4, 5], although some authors question the role and value of EDX testing in patients with suspected CTS [6, 7]. Additionally, high‐frequency ultrasound imaging (USI) has been shown to be a reliable, convenient, safe, and cost‐effective point‐of‐care imaging modality for a variety of neuromusculoskeletal and non‐neuromusculoskeletal conditions including evaluation of peripheral nerve morphology and perineural soft tissues, superficial tissues including breast, and vasculature [8, 9, 10, 11, 12, 13, 14]. Studies investigating the value of USI in patients with suspected CTS have identified median nerve cross‐sectional area (CSA) as being the most useful finding in the differential diagnostic process, although other median nerve characteristics have been evaluated in these patients including flattening ratio, transverse sliding, and hypervascularity [15, 16, 17, 18, 19, 20, 21, 22, 23, 24, 25, 26, 27, 28]. Recent studies have demonstrated the diagnostic value of measuring median nerve CSA at or near the wrist in the diagnosis of CTS and have provided updated recommendations for appropriate diagnostic cutoff values [29, 30]. Contemporary neuromuscular evaluation of patients with suspected CTS often includes *both* EDX testing and USI to inform the differential diagnostic process and guide clinical decision‐making by quantifying and characterizing peripheral nerve function and nerve morphology [9, 14, 20, 31].

Sonographic‐based measurements of median nerve CSA are typically performed in the distal wrist (dCSA) at or near the carpal tunnel inlet and proximal edge of the flexor retinaculum and sometimes in the proximal wrist (pCSA) at or near the level of the pronator quadratus muscle when the median nerve is positioned between the flexor digitorum superficialis and profundus tendons [23]. Additionally, some authors have recommended calculating the relative gauge change or delta of median nerve CSA (ΔCSA) by subtracting the pCSA measurement from the dCSA measurement, which in the absence of neuropathy should result in negative or near zero values [25, 27]. A recent study by Savage and McKell demonstrated that *binary* categorization of an USI diagnosis of CTS resulted in the best overall diagnostic accuracy based on likelihood ratios capable of producing meaningful shifts in diagnostic probability [29]. The authors recommended the following median nerve dCSA or ΔCSA cutoff values that can produce large conclusive shifts in diagnostic probability, which are useful regardless of disease prevalence or pretest probability: < 7 or < 1 mm^2^ to rule out and > 13 or > 7 mm^2^ to rule in CTS. Additionally, the authors recommended the following median nerve dCSA or ΔCSA cutoff values that produce only small questionable shifts in diagnostic probability, which are only useful when the disease prevalence or pretest probability is higher: ≤ 10 or ≤ 3 mm^2^ to rule out and ≥ 11 or ≥ 4 mm^2^ to rule in CTS [29].

Prior studies have demonstrated generally good agreement between EDX and USI diagnoses of CTS, but recognize that a percentage of limbs have divergent electrophysiologic and sonographic findings resulting in opposite diagnostic conclusions [23, 32, 33, 34]. This poses a challenge for clinicians that treat patients with suspected CTS because medical management decisions hinge on determining an appropriate diagnosis. Furthermore, clinicians utilize different EDX‐ and USI‐based criteria for evaluating median neuropathy at or near the wrist and therefore can arrive at very different conclusions. This problem is particularly keen for clinicians that do not utilize both EDX testing and USI in patients with suspected CTS because a negative result may not accurately diagnose a patient's condition and therefore result in inappropriate medical management [24, 35, 36, 37]. Previous studies in patients with a clinical suspicion of CTS have investigated sonographic findings in limbs with normal electrophysiologic findings, but the authors could find no studies investigating electrophysiologic findings in limbs with normal sonographic findings [24, 35, 36, 37, 38]. Additionally, the authors could find no studies evaluating the electrophysiologic and sonographic findings in limbs where the EDX and USI diagnoses of CTS diverged based on established diagnostic classifications systems.

The purpose of this investigation was to evaluate the electrophysiologic and sonographic findings in patients with suspected CTS that had divergent EDX and USI diagnoses of CTS. Specifically, using contemporary EDX‐ and USI‐based CTS classification systems, investigate limbs with normal electrophysiologic findings but a USI diagnosis of CTS and limbs with an EDX diagnosis of CTS but normal sonographic findings. Based on prior studies and clinical experience, the hypothesis of this investigation is that median nerve CSA will provide the most meaningful information when EDX and USI diagnoses of CTS diverge.

## 2 Materials and Methods

This was a retrospective cross‐sectional study design evaluating patient data where EDX and USI diagnoses of CTS diverged. All study‐related procedures were approved by the Institutional Review Board at Winston‐Salem State University (IRB‐FY2023‐3). Study participants were patients that underwent both EDX testing and USI in at least one limb for suspected CTS in the Department of Physical Therapy, Therapy West Physical Therapy & Sports Medicine Centers located in Richfield and Gunnison, Utah. Participating patients provided written informed consent to undergo testing, and every effort was made to ensure the rights of all participants were protected, including the handling of personal and health‐related information.

### 2.1 Electrodiagnostic Testing and Ultrasound Imaging

Electrodiagnostic testing was performed by two examiners, with the final EDX impression being determined for all limbs by the principal investigator, who is a Board‐Certified Clinical Electrophysiologic Specialist by the American Board of Physical Therapist Specialties and has over 18 years of experience performing, teaching, and publishing in the specialty area of clinical electrophysiology. Sierra Wave and Sierra Summit devices (Cadwell; Kennewick, WA, USA) were used for all examinations. Upper extremity nerve conduction studies were performed with patients seated and skin temperature maintained ≥ 32°C. Sensory and motor nerve conduction studies followed the standardized setup and performance described by Buschbacher, including analysis of distal latencies, conduction velocities, and amplitudes based on normative values stratified by patient sex and age [39]. Needle electromyography was performed with patients in supine using a monopolar needle electrode to evaluate insertional, resting, and volitional muscle activity, including analysis of motor unit recruitment patterns and morphology of individual motor unit potentials. Muscles analyzed represented the C5‐T1 myotomes and routinely included the deltoid, triceps, extensor carpi radialis, flexor carpi radialis, flexor carpi ulnaris, and first dorsal interosseous muscles. Based on a patient's signs and/or symptoms or specific nerve conduction findings, additional muscles were evaluated using needle electromyography when deemed appropriate and commonly included the abductor pollicis brevis or cervical paraspinal muscles. A final EDX impression was determined for all limbs and included the following categorizations of CTS severity based on the normative values described by Buschbacher: normal, mild, moderate, or severe. In general, a mild categorization involved sensory‐only findings (i.e., prolonged median sensory latency ≥ 4.0 ms to the middle finger or a prolonged median versus superficial radial sensory latency to the thumb); a moderate categorization involved sensory (i.e., absent or prolonged median sensory latency ≥ 4.0 ms to the middle finger) and motor findings (i.e., median motor latency ≥ 4.4 ms for women, ≥ 4.6 ms for men under 50 years, or ≥ 4.7 ms for men 50 years and older) and may have involved volitional electromyographic abnormalities; and a severe categorization involved absent sensory responses, prolonged or absent motor responses, and electromyographic evidence of axonopathy recorded in the abductor pollicis brevis muscle at rest.

Ultrasound imaging was performed by the principal investigator who is Registered in Musculoskeletal sonography by the Alliance for Physician Certification & Advancement with over 8 years of experience performing, teaching, and publishing in the specialty area of neuromusculoskeletal sonography. A high‐frequency 13–5 MHz linear transducer was used for all examinations (LOGIQ e BT12; GE HealthCare, Chicago, IL, USA). Patients were seated facing the examiner with their elbow extended, forearm supinated, and wrist/hand resting on a pillow in a neutral to slightly extended position. Short‐axis images were obtained of maximal median nerve pCSA at or near the level of the pronator quadratus muscle where the nerve is positioned between the flexor digitorum superficialis and profundus tendons, and maximal median nerve dCSA at or near the carpal tunnel inlet and proximal edge of the flexor retinaculum. Measurements of median nerve CSA were performed using an electronic caliper to trace the inner hyperechoic border of the epineurium.

### 2.2 Classification of Carpal Tunnel Syndrome Severity

**FIGURE 1:**
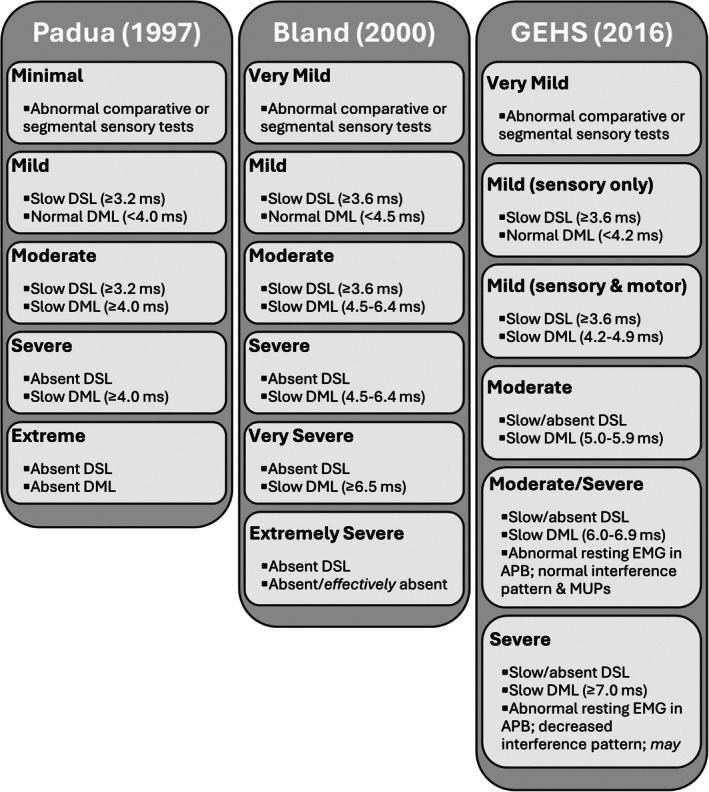
Summary of EDX‐based classifications of CTS severity. APB, abductor pollicis brevis; DMA, distal motor amplitude; DML, distal motor latency; DSL, distal sensory latency; EMG, electromyography; ms, milliseconds; MUP, motor unit potential; mV, millivolts.

Limbs were categorized according to CTS severity based on the final EDX impression and subsequently categorized according to the criteria described by Padua et al. [40], Bland [41], and Greathouse et al. (GEHS) [34] in their respective CTS severity classification systems (Figure 1). This approach was used to provide a meta‐analytic evaluation of divergent findings across multiple classification systems commonly used in clinical practice [42]. Notably, each of these EDX‐based classification systems utilizes different cutoff values for determining abnormalities in median nerve sensory and motor latencies, which differ from the normative values described by Buschbacher that are stratified based on sex and age categories and were used by the authors to determine the final EDX impression [39]. While the normative values recommended by Buschbacher differ from some of the criteria proposed by Padua, Bland, and GEHS, it should be understood that Buschbacher has not proposed a formal classification system [39]. Limbs not meeting the criteria for a CTS severity category within a particular classification system were placed in the category best fitting the electrophysiologic findings. For example, according to the Padua classification criteria, a limb with a sensory latency of 3.1 milliseconds and a motor latency of 5.5 milliseconds does not fit into “Moderate” or “Severe” categorizations. The authors chose to place that limb in the “Moderate” category because, while the motor latency was prolonged, the sensory latency was not only present but considered normal.

For the purposes of analysis, all limbs were *binarily* categorized as either normal or having an EDX or USI diagnosis of CTS. For a USI diagnosis of CTS, median nerve CSA cutoff values described by Savage and McKell were used (Table 1), which is the largest study to date examining the diagnostic accuracy of median nerve CSA in patients with suspected CTS and included 468 patients that contributed 665 limbs [25, 27, 28, 29]. The authors determined that median nerve dCSA or ΔCSA cutoff values to categorize *CTS severity* are not recommended and lack statistical support to produce large conclusive shifts in diagnostic probability based on small diagnostic likelihood ratios. Rather, consistent with the work of Ziswiler [28], *binary* categorization of CTS is recommended using median nerve dCSA or ΔCSA cutoff values that produce large conclusive shifts in diagnostic probability based on robust diagnostic likelihood ratios that can be used by clinicians regardless of disease prevalence or pretest probability. Additionally, the authors recommended median nerve dCSA or ΔCSA cutoff values that can be used in circumstances where the presence of CTS is deemed more likely based on a higher disease prevalence or pretest probability. These results were statistically robust and generally consistent across all EDX‐based classification systems examined, including the final EDX impression, Padua, Bland, and GEHS, providing a meta‐analytic approach to understanding shifts in diagnostic probabilities using contemporary evidence‐based median nerve CSA cutoff values [29].

**TABLE 1:** Distribution of limbs and associated diagnostic accuracy using recommended median nerve CSA cutoff values to rule out or rule a CTS diagnosis.

Median nerve CSA	Count (%)	CTS diagnosis	Shift in diagnostic probability
dCSA or ΔCSA			
< 7 or < 1 mm^2^	55 (8%)	Rule out	Large conclusive
> 13 or > 7 mm^2^	236 (36%)	Rule in	Large conclusive
Unclassified	347 (56%)	Equivocal	NA
dCSA or ΔCSA			
≤ 10 or ≤ 3 mm^2^	180 (27%)	Rule out	Small questionable
≥ 11 or ≥ 4 mm^2^	485 (73%)	Rule in	Small questionable

Abbreviations: ΔCSA, delta cross‐sectional area; CTS, carpal tunnel syndrome; dCSA, distal cross‐sectional area; mm^2^, millimeters squared; NA, not applicable.

### 2.3 Data Analysis

SPSS (IBM Statistics, version 28.0.1.0.; Armonk, NY, USA) was used for all data analysis. The data were screened for normality using the Kolmogorov–Smirnov test, histograms, and QQ plots. Descriptive statistics summarized characteristics of participating patients and limbs tested. MANOVA was used to compare variables in limbs with divergent *binary* EDX and USI diagnoses of CTS using Bonferroni corrections for multiple comparisons. Complete statistical analyses were confined to limbs categorized based on the final EDX impression. Dependent variables included patient age and height, median nerve sensory and motor latencies, median nerve motor amplitude, and median nerve CSA measurements. Independent variables included grouping limbs based on the categorization of EDX and USI diagnoses of CTS.

Chi Square analysis was used to evaluate the prevalence and distribution of limbs based on sex, age category, self‐reported diabetes diagnosis treated with medication, limb tested, electromyographic findings in the abductor pollicis brevis muscle, presence of bifid median nerve anatomy, and presence of volar wrist ganglion cyst organized by EDX and USI diagnoses of CTS. Bivariate Spearman correlation coefficients were calculated between median nerve CSA measurements, age, height, and all median nerve sensory and motor conduction variables organized by EDX and USI diagnoses of CTS.

## 3 Results

Data was collected from December 2019 through July 2023 on 468 patients (54.6 ± 16.8 years; 67.1 ± 3.9 in.; 59% female) referred for EDX testing and USI for suspected CTS (51% of patients were referred by a Physician Assistant or Nurse Practitioner; 49% of patients were referred by a Medical Doctor or Doctor of Osteopathy) that contributed 665 limbs (51% right). EDX testing was completed by two examiners (78% NJS; 22% JSM) and USI was completed by a single examiner (NJS). The prevalence of self‐reported diabetes diagnosis treated with medication was 17% (111 of 665 hands), the prevalence of bifid median nerve anatomy was 14% (92 of 665 hands), and the prevalence of volar wrist ganglion cyst was 44% (295 of 665 hands). To illustrate the differences in divergent electrophysiologic and sonographic findings across commonly used classification systems and the final EDX impression, the distribution of limbs based on binary EDX and USI diagnoses of CTS are found in Table 2. The following analyses are based exclusively on the binary final EDX impression, which utilized the normative values described by Buschbacher [39]. Data were not normally distributed and were unable to be successfully transformed using a square root transformation.

**TABLE 2:** Limbs with concordant and divergent EDX and USI diagnoses of CTS.

	−EDX and −USI	+EDX and +USI	Agree	−EDX and +USI	+EDX and −USI	Diverge
	*N*	*N*		*N*	*N*	
Large conclusive shifts in probability						
Final EDX impression	40	228	92.1%	8	15	7.9%
Padua classification	17	235	86.6%	1	38	13.4%
Bland classification	36	230	91.4%	6	19	8.6%
GEHS classification	34	230	90.7%	6	21	9.3%
Small questionable shifts in probability						
Final EDX impression	111	425	80.6%	60	69	19.4%
Padua classification	40	475	77.4%	10	140	22.6%
Bland classification	93	440	80.2%	45	87	19.8%
GEHS classification	91	442	80.2%	43	89	19.8%

Abbreviations: +EDX, electrodiagnostic diagnosis of CTS; −EDX, normal electrophysiologic findings; +USI, ultrasound imaging diagnosis of CTS; −USI, normal sonographic findings; CTS, carpal tunnel syndrome.

### 3.1 Final EDX Impression RULING OUT Carpal Tunnel Syndrome

For median nerve CSA cutoff values resulting in large conclusive shifts in diagnostic probability, the EDX and USI diagnoses of CTS were concordant in 40 out of 48 limbs (83%). Compared to the 8 limbs (17%) that had a USI diagnosis of CTS but normal electrophysiologic findings, MANOVA revealed significantly slower median nerve sensory latencies to the thumb and middle finger and significantly larger median nerve dCSA and ΔCSA, all with large effect sizes (Table 3). Chi Square analysis in limbs with normal electrophysiologic findings and concordant or divergent USI diagnoses of CTS revealed no significant differences in any variable tested. Significant weak correlations were found between demographic, electrophysiologic, and sonographic variables in limbs with a USI diagnosis of CTS but normal electrophysiologic findings, explaining between 13% and 20% of the variance.

**TABLE 3:** Limbs with concordant and divergent USI diagnoses of CTS with normal electrophysiologic findings.

	−EDX and −USI	−EDX and +USI	*p*	ƞ^2^
	Mean ± SD	Mean ± SD		
Large conclusive shifts in probability				
Age (years)	45.7 ± 16.3	47.2 ± 19.2	0.816	0.001
Height (inches)	65.8 ± 4.2	66.6 ± 3.5	0.587	0.006
DSL1 (ms)	2.6 ± 0.2	2.8 ± 0.1	*0.004*	*0.166*
DSL3 (ms)	3.2 ± 0.3	3.6 ± 0.2	*0.006*	*0.155*
DML (ms)	3.5 ± 0.4	3.7 ± 0.2	0.157	0.043
DMA (mV)	8.4 ± 2.9	8.9 ± 1.9	0.656	0.004
dCSA (mm^2^)	6.9 ± 1.8	15.6 ± 2.3	*< 0.001*	*0.754*
pCSA (mm^2^)	7.0 ± 2.1	7.4 ± 1.5	0.654	0.004
ΔCSA (mm^2^)	−0.1 ± 0.9	8.3 ± 1.6	*< 0.001*	*0.897*
Small questionable shifts in probability				
Age (years)	45.9 ± 14.5	49.0 ± 15.1	0.199	0.010
Height (inches)	66.4 ± 3.8	65.7 ± 2.8	0.169	0.011
DSL1 (ms)	2.6 ± 0.3	2.7 ± 0.2	*0.009*	*0.040*
DSL3 (ms)	3.3 ± 0.3	3.4 ± 0.3	*0.009*	*0.039*
DML (ms)	3.5 ± 0.4	3.6 ± 0.4	*0.040*	*0.025*
DMA (mV)	9.0 ± 3.1	8.4 ± 2.5	0.184	0.010
dCSA (mm^2^)	7.7 ± 1.4	11.1 ± 2.4	*< 0.001*	*0.451*
pCSA (mm^2^)	6.5 ± 1.4	6.1 ± 1.9	0.144	0.013
ΔCSA (mm^2^)	1.3 ± 1.3	5.1 ± 1.9	*< 0.001*	*0.574*

Abbreviations: ΔCSA, delta cross‐sectional area; ƞ^2^, partial eta squared; −EDX, normal electrophysiologic findings; +USI, ultrasound imaging diagnosis of CTS; −USI, normal sonographic findings; CTS; carpal tunnel syndrome; dCSA, distal cross‐sectional area; DML, distal motor latency; DSL1, distal sensory latency to thumb; DSL3, distal sensory latency to middle finger; mm^2^, millimeters squared; ms, milliseconds; mV, millivolts; pCSA, proximal cross‐sectional area; SD, standard deviation.

Similarly, for median nerve CSA cutoff values resulting in small questionable shifts in diagnostic probability, EDX and USI diagnoses of CTS were concordant in 111 out of 171 limbs (65%). Compared to the 60 limbs (35%) that had a USI diagnosis of CTS but normal electrophysiologic findings, MANOVA revealed significantly slower median nerve sensory latencies to the thumb and middle finger and significantly slower median nerve motor latency with small to medium effect sizes and significantly larger median nerve dCSA and ΔCSA with large effect sizes (Table 3). Chi Square analysis in limbs with normal electrophysiologic findings and concordant or divergent USI diagnoses of CTS revealed no significant differences in any variable tested. Significant weak correlations were found between demographic, electrophysiologic, and sonographic variables in limbs with a USI diagnosis of CTS but normal electrophysiologic findings explaining between 3% and 12% of the variance.

### 3.2 Final EDX Impression RULING IN Carpal Tunnel Syndrome

For median nerve CSA cutoff values resulting in large conclusive shifts in diagnostic probability, EDX and USI diagnoses of CTS were concordant in 228 out of 243 limbs (94%). Compared to the 15 limbs (6%) that had an EDX diagnosis of CTS but normal sonographic findings, MANOVA revealed significantly smaller median nerve dCSA and ΔCSA with large effect sizes and a significantly larger median nerve pCSA with a small effect size (Table 4). Chi Square analysis in limbs with an EDX diagnosis of CTS and concordant or divergent USI diagnosis of CTS revealed no significant differences in any variable tested.

**TABLE 4:** Limbs with concordant and divergent USI diagnoses of CTS with EDX diagnosis of CTS.

	+EDX and +USI	+EDX and −USI	*p*	ƞ^2^
	Mean ± SD	Mean ± SD		
Conclusive diagnostic accuracy				
Age (years)	56.0 ± 15.9	60.2 ± 20.6	0.339	0.004
Height (inches)	67.3 ± 3.9	67.1 ± 5.1	0.843	0.000
DSL1 (ms)	2.4 ± 1.9	2.8 ± 1.6	0.397	0.003
DSL3 (ms)	3.0 ± 2.4	3.5 ± 1.9	0.504	0.002
DML (ms)	5.6 ± 2.3	4.8 ± 0.9	0.186	0.007
DMA (mV)	5.9 ± 3.1	5.5 ± 2.0	0.566	0.001
dCSA (mm^2^)	17.4 ± 4.2	8.0 ± 1.5	*< 0.001*	*0.238*
pCSA (mm^2^)	7.2 ± 1.8	8.4 ± 1.9	*0.018*	*0.023*
ΔCSA (mm^2^)	10.1 ± 4.3	−0.4 ± 0.9	*< 0.001*	*0.272*
Questionable diagnostic accuracy				
Age (years)	57.1 ± 16.3	61.1 ± 16.3	0.066	0.007
Height (inches)	67.3 ± 3.9	66.9 ± 4.4	0.546	0.001
DSL1 (ms)	2.6 ± 1.8	2.9 ± 1.4	0.073	0.007
DSL3 (ms)	3.2 ± 2.2	3.6 ± 1.8	0.236	0.003
DML (ms)	5.4 ± 2.2	4.8 ± 1.5	*0.028*	*0.010*
DMA (mV)	6.1 ± 3.2	5.8 ± 2.8	0.502	0.001
dCSA (mm^2^)	14.6 ± 4.3	8.6 ± 1.2	*< 0.001*	*0.212*
pCSA (mm^2^)	6.9 ± 1.7	6.9 ± 1.4	0.891	0.000
ΔCSA (mm^2^)	7.7 ± 4.2	1.6 ± 1.3	*< 0.001*	*0.225*

Abbreviations: ΔCSA, delta cross‐sectional area; ƞ^2^, partial eta squared; −EDX, normal electrophysiologic findings; +USI, ultrasound imaging diagnosis of CTS; −USI, normal sonographic findings; CTS, carpal tunnel syndrome; dCSA, distal cross‐sectional area; DML, distal motor latency; DSL1, distal sensory latency to thumb; DSL3, distal sensory latency to middle finger; mm^2^, millimeters squared; ms, milliseconds; mV, millivolts; pCSA, proximal cross‐sectional area; SD, standard deviation.

Comparison of limbs with and without an USI diagnosis of CTS with severity categorizations in the final EDX impression (i.e., mild, moderate, or severe) found significant differences in limbs with divergent EDX and USI diagnoses of CTS in the moderate and severe categorizations, with prevalences ranging from a high of 67% to a low of 0%, respectively (χ^2^ = 9.1, *p* = 0.010). Significant weak correlations were found between demographic, electrophysiologic, and sonographic variables in limbs with an EDX diagnosis of CTS but normal sonographic findings, explaining between 2% and 12% of the variance.

For median nerve CSA cutoff values resulting in small questionable shifts in diagnostic probability, EDX and USI diagnoses of CTS were concordant in 111 out of 171 limbs (65%). Compared to the 60 limbs (35%) that had an EDX diagnosis of CTS but normal sonographic findings, MANOVA revealed significantly faster median nerve motor latency with a small effect size and significantly larger median nerve dCSA and ΔCSA with large effect sizes (Table 4). Chi Square analysis in limbs with an EDX diagnosis of CTS and concordant or divergent USI diagnoses of CTS revealed no significant differences in any variables tested. Comparison of limbs with and without an USI diagnosis of CTS with severity categorizations in the final EDX impression (i.e., mild, moderate, or severe) found significant differences in limbs with divergent EDX and USI diagnoses of CTS in the moderate and severe categorizations with prevalences ranging from a high of 58% to a low of 12%, respectively (χ^2^ = 8.4, *p =* 0.015). Significant weak correlations were found between demographic, electrophysiologic, and sonographic variables in limbs with an EDX diagnosis of CTS but normal sonographic findings, explaining between 1% and 7% of the variance.

### 3.3 Additional Analysis

Because of the known impact on median nerve CSA, an analysis was conducted excluding limbs from patients that either had bifid median nerve anatomy or self‐reported diabetes diagnosis treated with medication [43, 44]. While the exclusion of these limbs resulted in generally less divergent findings (Table 1A, Appendix A), the analysis did not alter any outcomes or conclusions of the study and outside of the change in a single variable (i.e., DML in Table 3A, Appendix A) did not alter any significance or effect size values (Tables 2A and 3A, Appendix A).

To evaluate possible violations of the assumption of statistical independence because some patients contributed both limbs, an analysis was conducted that included only one limb from each patient. While the inclusion of a single limb resulted in generally more divergent findings (Tables 1B, Appendix B), the analysis did not alter any outcomes or conclusions of the study and did not alter any significance or effect size values (Tables 2B and 3B, Appendix B).

## 4 Discussion

The most common divergent findings in this investigation were limbs with an EDX diagnosis of CTS but normal sonographic findings. This study evaluated limbs with normal electrophysiologic findings but an USI diagnosis of CTS and limbs with an EDX diagnosis of CTS but normal sonographic findings. Although the distribution of limbs in this study revealed generally good concordance between EDX and USI diagnoses of CTS regardless of the EDX‐based classification system evaluated, these findings provide novel data evaluating the electrophysiologic and sonographic findings in limbs where the results of these point‐of‐care diagnostic tools diverge. The prevalence of divergent EDX and USI diagnoses of CTS increased as diagnostic accuracy decreased, namely going from a criterion that produces large conclusive shifts to a criterion that produces only small questionable shifts in diagnostic probability. Although this finding is not surprising, it is notable because providers in clinical practice commonly use a variety of median nerve CSA cutoff values, most of which produce only small questionable shifts in diagnostic probability and therefore result in increased diagnostic uncertainty [23, 27, 28]. Additionally, these findings highlight the fact that the median nerve CSA cutoff values used by clinicians to determine an USI diagnosis of CTS have a direct impact on the prevalence of divergent findings regardless of the EDX‐based classification system used, which directly informs medical management decisions in these patients [29, 42].

Consistent with the results of this investigation, a recent study by Chen et al. investigating sonographic findings in 24 patients contributing 28 limbs with a clinical diagnosis of CTS but normal nerve conduction found that median nerve dCSA was significantly larger compared to 42 healthy controls contributing 52 limbs. The authors concluded that USI has unique diagnostic value in these patients [35]. Similarly, Akturk et al. investigated sonographic findings in 41 patients contributing 51 limbs with a clinical diagnosis of CTS but normal nerve conduction and found significantly larger median nerve dCSA, decreased echogenicity, and decreased mobility compared to 20 healthy controls contributing 30 limbs. The authors found significant correlations between median nerve dCSA and clinically measured sensory loss, symptom provocation tests, body mass index, and the Boston Carpal Tunnel Questionnaire scores, concluding that USI helps clinicians diagnose CTS when electrophysiologic findings are normal [36]. Aseem et al. investigated sonographic findings in the median nerve in 14 patients contributing 22 limbs with a clinical diagnosis of CTS but normal nerve conduction and found 92% had enlarged dCSA, 100% had increased wrist‐to‐forearm versus median nerve area ratios, 82% had decreased echogenicity, 75% had decreased mobility, and 7% had increased vascularity. The authors concluded that a large proportion of patients with a clinical suspicion of CTS but normal electrophysiologic findings have abnormal sonographic findings suggesting a CTS diagnosis [37]. Finally, Al‐Hashel et al. investigated sonographic findings in 35 patients contributing 60 limbs with a clinical diagnosis of CTS but normal nerve conduction and found 49% had increased median nerve dCSA and 34% had an increased thickness of the flexor retinaculum compared to 20 healthy controls contributing 40 limbs. The authors found abnormal median nerve dCSA was correlated with clinical measurements of sensory loss and positive provocation tests and concluded that the clinical and USI diagnoses of CTS converged in about half of the patients that had normal electrophysiologic findings [38]. In contrast to prior studies investigating sonographic findings in patients with a clinical diagnosis of CTS but normal electrophysiologic findings, the current investigation included an analysis of patients with an EDX diagnosis of CTS but normal sonographic findings. Additionally, prior studies utilized multiple t‐tests in their analyses and did not describe how they corrected for an inflated familywise error rate (i.e., type 1 statistical error), something which this study accounted for by employing robust statistical techniques including MANOVA and Bonferroni corrections.

This investigation found significantly slower median nerve sensory and motor latencies in hands with a USI diagnosis of CTS but normal electrophysiologic findings; however, these differences are not considered clinically meaningful based on the published criteria of most EDX‐based CTS severity classification systems [34, 39, 41]. The lone exception being the median nerve sensory latency criteria described by Padua [40]. In contrast, the significantly larger median nerve dCSA and ΔCSA found in limbs with a USI diagnosis of CTS but normal electrophysiologic findings are considered clinically meaningful cutoff values by most authors [15, 16, 17, 18, 19, 21, 22, 23, 25, 27, 28, 33]. The significantly smaller median nerve pCSA found in limbs with a USI diagnosis of CTS in the large conclusive diagnostic accuracy group had a small effect size and is unlikely to be clinically meaningful. Additionally, the correlations between demographic, electrophysiologic, and sonographic variables were generally weak and not considered clinically meaningful, explaining less than 10% of variance in nearly every instance.

This study provides novel data evaluating electrophysiologic and sonographic findings in limbs with divergent EDX and USI diagnoses of CTS, particularly the analysis of limbs with an EDX diagnosis of CTS but normal sonographic findings. This study is also unique by considering divergent EDX and USI diagnoses of CTS across commonly used EDX‐ and USI‐based classification systems, a meta‐analytic approach allowing the framing of the findings in the context of diagnostic accuracy and strengthening the generalizability and clinical utility of the results. These findings will better inform clinicians specializing in neuromusculoskeletal medicine by improving evaluation, differential diagnosis, and medical management of patients with suspected CTS.

The authors acknowledge some limitations of this study. First, USI was performed by a single examiner who was not blinded to most of the electrophysiologic findings, having performed 78% of the EDX tests, which may have introduced error in median nerve CSA measurements. Alternatively, having a single examiner perform all USI improves the internal reliability of our study and is most likely to result in more agreement and fewer divergent findings. Second, the authors accepted patient self‐reported diagnosis of diabetes treated with medication rather than laboratory confirmation; while this approach is consistent with clinical practice, it may have introduced variability in median nerve CSA, as some of these conditions can significantly impact peripheral nerve health and morphology. However, the presence of subclinical pathology is less likely to result in significant physiological changes and therefore less likely to have a meaningful impact on peripheral nerve function and/or morphology.

## 5 Conclusions

The results of this study confirmed the hypothesis that median nerve CSA provides the most meaningful information when the EDX and USI diagnoses of CTS diverge in patients with a clinical suspicion of CTS. This conclusion is based on the observation that in limbs with divergent electrophysiologic and sonographic findings, differences in median nerve CSA were clinically significant (i.e., clearly abnormal based on best available diagnostic criteria) with large effect sizes. In contrast, differences in sensory and motor nerve conduction, while statistically significant in some instances, were not considered clinically meaningful based on most published normative criteria and all had small effect sizes. This study underscores the value of integrating EDX testing and USI in patients with suspected CTS, particularly when considering the reality of divergent diagnoses when evaluating the findings from independent diagnostic tests. While EDX testing remains the gold standard test for evaluating peripheral nerve function, USI has increasingly become an indispensable point‐of‐care diagnostic tool capable of providing unique structural and morphological information about peripheral nerves. The combination of these point‐of‐care diagnostic tools in the hands of a neuromusculoskeletal specialist provides superior diagnostic accuracy when compared to relying on either alone. Future research should focus on optimizing protocols integrating EDX testing and USI to enhance diagnostic precision, refine the differential diagnostic process, and improve medical management decisions related to neuromusculoskeletal disorders in routine clinical practice.

## Disclosure

All study procedures were approved for use in human subjects by the Institutional Review Board at Winston‐Salem State University (IRB‐FY2024‐7).

## Conflicts of Interest

The authors declare no conflicts of interest.

## Data Availability

The data that support the findings of this study are available from the corresponding author upon reasonable request.

## Appendix A

*Analysis *excludes limbs with bifid median nerve anatomy or* from patients that reported taking medication for a *diabetes diagnosis*.

**TABLE 1A:** Limbs with concordant and divergent EDX and USI diagnoses of CTS.

	−EDX and −USI	+EDX and +USI	Agree	−EDX and +USI	+EDX and −USI	Diverge
	*N*	*N*		*N*	*N*	
Large conclusive shifts in probability						
Final EDX impression	28	168	93.8%	5	8	6.2%
Padua classification	13	172	88.5%	1	23	11.5%
Bland classification	27	168	93.3%	5	9	6.7%
GEHS classification	26	168	92.8%	5	10	7.2%
Small questionable shifts in probability						
Final EDX impression	82	299	80.4%	46	47	19.6%
Padua classification	29	337	77.2%	8	100	22.8%
Bland classification	71	309	80.2%	36	58	19.8%
GEHS classification	70	311	80.4%	34	59	19.6%

Abbreviations: +EDX, electrodiagnostic diagnosis of CTS; −EDX, normal electrophysiologic findings; +USI, ultrasound imaging diagnosis of CTS; −USI, normal sonographic findings; CTS, carpal tunnel syndrome.

**TABLE 2A:** Limbs with concordant and divergent USI diagnoses of CTS with normal electrophysiologic findings.

	−EDX and −USI	−EDX and +USI	*p*	ƞ^2^
	Mean ± SD	Mean ± SD		
Large conclusive shifts in probability				
Age (years)	45.4 ± 17.1	41.5 ± 19.1	0.649	0.007
Height (inches)	66.3 ± 4.4	65.4 ± 2.5	0.682	0.005
DSL1 (ms)	2.6 ± 0.3	2.9 ± 0.1	0.017	0.170
DSL3 (ms)	3.2 ± 0.3	3.6 ± 0.2	0.026	0.150
DML (ms)	3.5 ± 0.4	3.7 ± 0.2	0.319	0.032
DMA (mV)	8.7 ± 3.2	8.9 ± 1.9	0.927	0.000
dCSA (mm^2^)	6.7 ± 1.6	15.2 ± 1.9	< 0.001	0.788
pCSA (mm^2^)	6.5 ± 2.1	6.8 ± 1.3	0.785	0.002
ΔCSA (mm^2^)	0.1 ± 0.8	8.4 ± 1.5	< 0.001	0.910
Small questionable shifts in probability				
Age (years)	44.7 ± 14.0	47.9 ± 15.4	0.240	0.011
Height (inches)	66.7 ± 3.7	65.6 ± 2.7	0.075	0.025
DSL1 (ms)	2.6 ± 0.3	2.7 ± 0.2	0.013	0.048
DSL3 (ms)	3.3 ± 0.3	3.4 ± 0.3	0.008	0.054
DML (ms)	3.5 ± 0.4	3.6 ± 0.4	0.021	0.041
DMA (mV)	9.3 ± 3.3	8.3 ± 2.5	0.073	0.025
dCSA (mm^2^)	7.7 ± 1.4	11.1 ± 1.9	< 0.001	0.517
pCSA (mm^2^)	6.3 ± 1.3	5.9 ± 1.7	0.276	0.009
ΔCSA (mm^2^)	1.4 ± 1.2	5.1 ± 1.8	< 0.001	0.612

Abbreviations: ΔCSA, delta cross‐sectional area; ƞ^2^, partial eta squared; −EDX, normal electrophysiologic findings; +USI, ultrasound imaging diagnosis of CTS; −USI, normal sonographic findings; CTS; carpal tunnel syndrome; dCSA, distal cross‐sectional area; DML, distal motor latency; DSL1, distal sensory latency to thumb; DSL3, distal sensory latency to middle finger; mm^2^, millimeters squared; ms, milliseconds; mV, millivolts; pCSA, proximal cross‐sectional area; SD, standard deviation.

**TABLE 3A:** Limbs with concordant and divergent USI diagnoses of CTS with EDX diagnosis of CTS.

	+EDX and +USI	+EDX and −USI	*p*	ƞ^2^
	Mean ± SD	Mean ± SD		
Conclusive diagnostic accuracy				
Age (years)	54.4 ± 16.7	54.9 ± 27.2	0.940	0.000
Height (inches)	67.0 ± 4.1	66.0 ± 5.9	0.509	0.003
DSL1 (ms)	2.6 ± 1.9	3.3 ± 1.6	0.291	0.006
DSL3 (ms)	3.1 ± 2.4	3.9 ± 1.9	0.349	0.005
DML (ms)	5.7 ± 2.3	4.9 ± 1.2	0.363	0.005
DMA (mV)	5.9 ± 3.0	6.5 ± 1.7	0.607	0.005
dCSA (mm^2^)	17.1 ± 3.8	8.3 ± 1.8	< 0.001	0.002
pCSA (mm^2^)	7.1 ± 1.8	8.5 ± 2.6	0.033	0.200
ΔCSA (mm^2^)	10.0 ± 3.9	−0.3 ± 1.0	< 0.001	0.026
				0.242
Questionable diagnostic accuracy				
Age (years)	55.1 ± 16.9	59.6 ± 17.8	0.097	0.008
Height (inches)	66.9 ± 3.9	66.2 ± 4.4	0.227	0.004
DSL1 (ms)	2.7 ± 1.8	3.2 ± 1.2	0.064	0.010
DSL3 (ms)	3.3 ± 2.2	3.9 ± 1.5	0.067	0.010
DML (ms)	5.5 ± 2.2	5.1 ± 1.4	0.287	0.003
DMA (mV)	6.1 ± 3.1	6.1 ± 2.9	0.985	0.000
dCSA (mm^2^)	14.6 ± 4.1	8.7 ± 1.2	< 0.001	0.216
pCSA (mm^2^)	6.7 ± 1.7	6.9 ± 1.5	0.608	0.001
ΔCSA (mm^2^)	7.8 ± 3.9	1.8 ± 1.2	< 0.001	0.239

Abbreviations: ΔCSA, delta cross‐sectional area; ƞ^2^, partial eta squared; −EDX, normal electrophysiologic findings; +USI, ultrasound imaging diagnosis of CTS; −USI, normal sonographic findings; CTS; carpal tunnel syndrome; dCSA, distal cross‐sectional area; DML, distal motor latency; DSL1, distal sensory latency to thumb; DSL3, distal sensory latency to middle finger; mm^2^, millimeters squared; ms, milliseconds; mV, millivolts; pCSA, proximal cross‐sectional area; SD, standard deviation.

## Appendix B

*Analysis that *includes contribution of a single limb* from each participant *to evaluate for the possible violation of the independence of observation principle* in statistical analysis.

**TABLE 1B:** Limbs with concordant and divergent EDX and USI diagnoses of CTS.

	−EDX and −USI	+EDX and +USI	Agree	−EDX and +USI	+EDX and −USI	Diverge
	*N*	*N*		*N*	*N*	
Large conclusive shifts in probability						
Final EDX impression	37	154	91.4%	6	12	8.6%
Padua classification	16	160	84.2%	0	33	15.8%
Bland classification	34	156	90.9%	4	15	9.1%
GEHS classification	32	156	90.0%	4	17	10.0%
Small questionable shifts in probability						
Final EDX impression	93	283	80.3%	41	51	19.7%
Padua classification	35	317	75.2%	7	109	24.8%
Bland classification	79	295	79.9%	29	65	20.1%
GEHS classification	77	296	79.7%	28	67	20.3%

Abbreviations: +EDX, electrodiagnostic diagnosis of CTS; −EDX, normal electrophysiologic findings; +USI, ultrasound imaging diagnosis of CTS; −USI, normal sonographic findings; CTS, carpal tunnel syndrome.

**TABLE 2B:** Limbs with concordant and divergent USI diagnoses of CTS with normal electrophysiologic findings.

	−EDX and −USI	−EDX and +USI	*p*	ƞ^2^
	Mean ± SD	Mean ± SD		
Large conclusive shifts in probability				
Age (years)	45.1 ± 16.4	46.7 ± 21.3	0.831	0.001
Height (inches)	65.7 ± 4.3	65.2 ± 2.3	0.782	0.002
DSL1 (ms)	2.5 ± 0.2	2.8 ± 0.1	0.006	0.171
DSL3 (ms)	3.2 ± 0.3	3.6 ± 0.2	0.013	0.142
DML (ms)	3.5 ± 0.4	3.7 ± 0.2	0.211	0.038
DMA (mV)	8.4 ± 3.1	8.4 ± 2.1	0.984	0.000
dCSA (mm^2^)	6.9 ± 1.8	16.0 ± 2.6	< 0.001	0.738
pCSA (mm^2^)	7.0 ± 2.1	7.3 ± 1.8	0.713	0.003
ΔCSA (mm^2^)	−0.0 ± 0.9	8.7 ± 1.5	< 0.001	0.902
Small questionable shifts in probability				
Age (years)	45.0 ± 14.5	48.9 ± 14.9	0.162	0.015
Height (inches)	66.5 ± 3.9	65.9 ± 2.8	0.400	0.005
DSL1 (ms)	2.6 ± 0.3	2.7 ± 0.2	0.026	0.037
DSL3 (ms)	3.3 ± 0.3	3.4 ± 0.3	0.035	0.033
DML (ms)	3.5 ± 0.4	3.6 ± 0.4	0.047	0.030
DMA (mV)	9.2 ± 3.2	8.3 ± 2.6	0.126	0.018
dCSA (mm^2^)	7.7 ± 1.4	11.4 ± 2.6	< 0.001	0.456
pCSA (mm^2^)	6.5 ± 1.4	6.3 ± 1.9	0.550	0.003
ΔCSA (mm^2^)	1.2 ± 1.3	5.1 ± 2.2	< 0.001	0.548

Abbreviations: ΔCSA, delta cross‐sectional area; ƞ^2^, partial eta squared; −EDX, normal electrophysiologic findings; +USI, ultrasound imaging diagnosis of CTS; −USI, normal sonographic findings; CTS, carpal tunnel syndrome; dCSA, distal cross‐sectional area; DML, distal motor latency; DSL1, distal sensory latency to thumb; DSL3, distal sensory latency to middle finger; mm^2^, millimeters squared; ms, milliseconds; mV, millivolts; pCSA, proximal cross‐sectional area; SD, standard deviation.

**TABLE 3B:** Limbs with concordant and divergent USI diagnoses of CTS with EDX diagnosis of CTS.

	+EDX and +USI	+EDX and −USI	*p*	ƞ^2^
	Mean ± SD	Mean ± SD		
Conclusive diagnostic accuracy				
Age (years)	56.7 ± 16.3	60.8 ± 19.0	0.400	0.004
Height (inches)	67.3 ± 3.9	67.2 ± 5.5	0.948	0.000
DSL1 (ms)	2.5 ± 2.0	2.8 ± 1.4	0.588	0.002
DSL3 (ms)	3.1 ± 2.5	3.4 ± 1.7	0.725	0.001
DML (ms)	5.6 ± 2.6	4.5 ± 0.7	0.135	0.014
DMA (mV)	5.9 ± 3.2	5.1 ± 2.0	0.416	0.004
dCSA (mm^2^)	17.2 ± 4.1	8.2 ± 1.5	< 0.001	0.264
pCSA (mm^2^)	7.2 ± 1.8	8.5 ± 1.7	0.019	0.033
ΔCSA (mm^2^)	9.9 ± 4.1	−0.3 ± 0.8	< 0.001	0.313
Questionable diagnostic accuracy				
Age (years)	57.4 ± 16.4	60.8 ± 16.1	0.176	0.005
Height (inches)	67.4 ± 3.9	67.1 ± 4.5	0.661	0.001
DSL1 (ms)	2.6 ± 1.8	3.1 ± 1.1	0.089	0.009
DSL3 (ms)	3.3 ± 2.3	3.6 ± 1.6	0.283	0.003
DML (ms)	5.4 ± 2.3	4.7 ± 1.2	0.042	0.012
DMA (mV)	6.1 ± 3.3	5.9 ± 2.8	0.663	0.001
dCSA (mm^2^)	14.6 ± 4.3	8.6 ± 1.2	< 0.001	0.231
pCSA (mm^2^)	6.9 ± 1.7	6.9 ± 1.4	0.866	0.000
ΔCSA (mm^2^)	7.7 ± 4.0	1.7 ± 1.3	< 0.001	0.246

Abbreviations: ΔCSA, delta cross‐sectional area; ƞ^2^, partial eta squared; −EDX, normal electrophysiologic findings; +USI, ultrasound imaging diagnosis of CTS; −USI, normal sonographic findings; CTS; carpal tunnel syndrome; dCSA, distal cross‐sectional area; DML, distal motor latency; DSL1, distal sensory latency to thumb; DSL3, distal sensory latency to middle finger; mm^2^, millimeters squared; ms, milliseconds; mV, millivolts; pCSA, proximal cross‐sectional area; SD, standard deviation.
